# The impact of the Adolescent Girls Empowerment Program (AGEP) on short and long term social, economic, education and fertility outcomes: a cluster randomized controlled trial in Zambia

**DOI:** 10.1186/s12889-020-08468-0

**Published:** 2020-03-17

**Authors:** Karen Austrian, Erica Soler-Hampejsek, Jere R. Behrman, Jean Digitale, Natalie Jackson Hachonda, Maximillian Bweupe, Paul C. Hewett

**Affiliations:** 1 Population Council, Nairobi, Kenya; 2 Independent Consultant, Barcelona, Spain; 3 University of Pennsylvania, Department of Economics, Philadelphia, Pennsylvania USA; 4 University of California, San Francisco, Deparment of Epidemiology and Biostatistics, San Francisco, California USA; 5 Population Council, Lusaka, Zambia; 6 Ministry of Health, Government of Republic of Zambia, Lusaka, Zambia; 7 Population Council, Washington, D.C., USA

**Keywords:** Adolescent girls, Randomized controlled trial, Zambia, Empowerment, Sexual and reproductive health, Safe spaces

## Abstract

**Background:**

Adolescent girls in Zambia face risks and vulnerabilities that challenge their healthy development into young women: early marriage and childbearing, sexual and gender-based violence, unintended pregnancy and HIV. The Adolescent Girls Empowerment Program (AGEP) was designed to address these challenges by building girls’ social, health and economic assets in the short term and improving sexual behavior, early marriage, pregnancy and education in the longer term. The two-year intervention included weekly, mentor-led, girls group meetings on health, life skills and financial education. Additional intervention components included a health voucher redeemable for general wellness and reproductive health services and an adolescent-friendly savings account.

**Methods:**

A cluster-randomized-controlled trial with longitudinal observations evaluated the impact of AGEP on key indicators immediately and two years after program end. Baseline data were collected from never-married adolescent girls in 120 intervention clusters (3515 girls) and 40 control clusters (1146 girls) and again two and four years later. An intent-to-treat analysis assessed the impact of AGEP on girls’ social, health and economic assets, sexual behaviors, education and fertility outcomes. A treatment-on-the-treated analysis using two-stage, instrumental variables regression was also conducted to assess program impact for those who participated.

**Results:**

The intervention had modest, positive impacts on sexual and reproductive health knowledge after two and four years, financial literacy after two years, savings behavior after two and four years, self-efficacy after four years and transactional sex after two and four years. There was no effect of AGEP on the primary education or fertility outcomes, nor on norms regarding gender equity, acceptability of intimate partner violence and HIV knowledge.

**Conclusions:**

Although the intervention led to sustained change in a small number of individual outcomes, overall, the intervention did not lead to girls acquiring a comprehensive set of social, health and economic assets, or change their educational and fertility outcomes. It is important to explore additional interventions that may be needed for the most vulnerable girls, particularly those that address household economic conditions. Additional attention should be given to the social and economic environment in which girls are living.

**Trial registration:**

ISRCTN29322231. Trial Registration Date: March 04, 2016; retrospectively registered.

## Background

Adolescent girls^1^ in Zambia face risks and vulnerabilities that challenge their healthy development into young women including early marriage and childbearing, sexual- and gender-based violence, unwanted pregnancy and the acquisition of HIV and other sexually-transmitted infections [1]. As indicated by the 2013–2014 Zambia Demographic and Health Survey (DHS) data, nearly one in three girls aged 20–24 had married by age 18, with a similar percentage having begun childbearing [2]. More than 35% of 15–24-year-old females have experienced physical violence, 12% have experienced sexual violence and 48% report that wife-beating is justified in certain circumstances [2]. Among 15–19-year-olds, 4.8% of females are HIV-positive as compared to 4.1% of males. The gender disparity increases in the 20–24-year-old group as 11.2% of females are HIV-positive as compared to 7.3% of males [2]. The literature shows that adolescent girls, particularly in developing countries, lack the assets and skills needed to break out of the cycle of poverty and capitalize on opportunities that exist [3, 4]. Furthermore, in these settings, women and girls’ opportunities are limited by traditional practices, adverse gender norms and roles and weak institutions and laws [3, 4]. For instance, Duflo argues that inequality, poverty and the lack of access to economic assets, opportunities and labor markets are primary drivers for the persistent disadvantage of women relative to men [3]. Other studies have directly linked gender power inequality to HIV risk behaviors and exposures [5]. For example, data from adolescent girls in South Africa showed that decreases in social isolation and economic vulnerability are linked to decreases in experiences of sexual coercion and transactional sex [6]. Calls have been made to address women’s and girls’ health issues via improvements in gender equality and empowerment [7, 8].

The Adolescent Girls Empowerment Program (AGEP) in Zambia hypothesized that one way to improve girls’ longer-term outcomes was through a program that aimed to empower adolescent girls by building their social, health and economic assets, allowing them to, in turn, reduce their vulnerabilities and capitalize on opportunities to improve their health, fertility and educational outcomes [1, 9, 10]. Studies have shown that some of these links between asset building and longer-term health and educational outcomes are present and therefore they are important to explore as pathways to health behavioral change. For example, a program in Uganda found that girls who had increased their social and health assets were able to simultaneously increase their economic assets without experiencing an increase in sexual harassment and violence as compared to girls who only built economic assets and experienced an increase in sexual harassment and violence as a result [11]. In Kenya, a qualitative study of young women 18–24 years old found that having savings was perceived as a key facilitator to translating knowledge on safer sex practices into practice [12]. This paper evaluates AGEP’s theory of change by assessing the impact of the program on adolescent girls’ mid- and longer-term indicators using data collected at the end of the two-year intervention, and 2 years after the end of the intervention.

## Methods

### Impact evaluation design

The AGEP evaluation was based on a multi-arm randomized cluster design implemented in ten sites, half urban and half rural, in four provinces in Zambia. Study provinces and the number of sites per province were selected purposefully, based on the feasibility of operating the program in the context of conducting an embedded randomized impact evaluation, as well as through discussions with the donor regarding the diversity of the target populations. Sites within provinces and urban/rural stratification, were randomly selected from a larger number of possible locations. Sites in rural areas contained multiple contiguous or proximal villages or chiefdoms, while in urban sites the program was implemented in one or more high-density housing compounds. The study adhered to CONSORT guidelines.

### Interventions and study arms

AGEP included three intervention components: The first was weekly girls group meetings that were facilitated by a female mentor from the community. Mentors were aged 20–35, selected via an interview process and participated in an initial 10-day training on the content and mentor skills, a five-day refresher training midway through the intervention and monthly supervision meetings with program staff. Groups were segmented by age and marital status, and included curricula-guided sessions on sexual and reproductive health, HIV, life skills and financial education. The weekly girls groups, known as ‘safe spaces,’ were considered the core component of AGEP as there was a growing literature that girls meeting regularly for sessions facilitated by a female mentor, combining sexual and reproductive health, life skills and economic-strengthening components—had been successful in several countries in sub-Saharan Africa for improving longer-term health and economic outcomes [11, 13, 14]. A second component of the intervention was the provision of a health voucher to girls, which could be used at contracted public and private facilities for free general wellness and sexual and reproductive health services. A third component of AGEP was the provision of an adolescent-friendly savings account, with features such as low fees, low opening balance (~$0.25) and the ability for a minor to transact on the account. The design of the bank accounts was developed in partnership with the National Savings and Credit Bank of Zambia. Further details of AGEP’s interventions are described elsewhere [1]. The study included three intervention arms to assess the effect of the weekly meetings alone, as well as the added effect of the “add-on components”: Arm 1 included weekly meetings only, Arm 2 included weekly meetings and the health voucher, and Arm 3 included weekly meetings, the health voucher and the savings account. A fourth arm was a control in which there were no interventions offered.

### Sample size and power analysis

A minimum detectable effect (MDE) approach was used to conduct power analysis as limited baseline evidence was available to support a sample size estimation approach. MDEs for the study’s longer-term outcomes comparing each arm against the control were estimated using Optimal Design Plus Software Version 3.0 for a multi-site cluster randomized trial [15]. The MDE estimation was stratified by urban/rural location and by younger and older cohorts (10–14 and 15–19). Endline (four years after the baseline) estimates of study indicators for the control arm were obtained from the 2007 Zambia DHS [16]. Power was set at 0.80, significance level at 0.05, and the effect size variability was fixed at 0.00, as analyses controlled for site fixed effects. Given the budget available for fieldwork and a total of ten study sites, MDEs for different combinations of numbers of clusters and respondents per cluster were compared and it was determined that the most efficient sample size was 40 clusters per study arm (four clusters per arm per site) and 20 participants per cluster, 10 per age cohort, for a total sample at endline of 3200. A cluster was defined as a Census Supervisory Area (CSA), as delineated by the Zambia Central Statistical Office.^2^ The MDEs are reported elsewhere [1].

### Randomization and study participants

Random selection of clusters and assignment to study arms was conducted at the site level via a public lottery attended by key community stakeholders. At the lottery, one bowl contained slips of papers that had one CSA code per slip and another bowl had sixteen slips of paper, each with one arm listed (four per arm). Representatives from the community came to the front, selected one CSA slip and then one study arm slip. The process was repeated 16 times, completing CSA selection and study arm assignment. Following the public lottery, a household listing was conducted in 2013 in all selected clusters. In service of the AGEP goal of reaching the most vulnerable girls, while also creating conditions for conducting a rigorous cluster randomized evaluation, eligible girls were identified for recruitment into the AGEP intervention through the household listing. A vulnerability score was assigned using an ordinary least squares (OLS) regression to estimate the number of grades behind for age as the dependent variable; independent variables in the regression model included age, not in school, ever married and having at least one child. The estimated residual of the regression was then used to represent vulnerability, with higher residuals indicating higher vulnerability. Both married and unmarried girls were ranked from highest to lowest vulnerability in each site and the 2000 most vulnerable in each site were tagged. Of tagged girls, those living in AGEP intervention clusters were invited to participate in the program, while girls living in control clusters were not issued an invitation.

The lists of most vulnerable girls in each site, excluding ever-married girls, constituted the sampling frame for the research. The baseline target sample was inflated to account for conservative estimates of attrition and refusals, in particular among the older cohort. Up to 17 girls aged 10–14 and up to 23 girls aged 15–19 within each cluster, stratified by two age groups (10–14 and 15–19 years old), were randomly selected for participation in the research.^3^

The AGEP research protocol was reviewed and approved by the Population Council Institutional Review Board (PC-IRB), the University of Zambia’s Research Ethics Committee (UNZA-REC) and the Zambian Ministry of Health. Informed consent was obtained from all participants. Parental or guardian consent was also obtained for participants under 18 years old.^4^

### Study instruments

The adolescent survey instruments were designed to measure both girls’ assets – including gender norms, self-efficacy, HIV and sexual and reproductive health (SRH) knowledge, financial literacy and savings behavior – as well as longer-term outcomes – including schooling attainment, age of sexual debut, first birth and marriage. Attendance data for each girl, including date of attendance and session topic, were collected on mobile phones by group mentors via Open Data Kit (ODK). Attendance data collected from ODK were then uploaded into a cloud-based database hosted on a cloud-based platform.

### Outcomes

This paper focuses on longer-term educational and fertility outcomes as well as a subset of hypothesized mediating indicators representing girls’ assets within each domain (social, health and economic). Additional file 1 provides detailed definitions for all outcomes and mediating indicators. Indicators were selected based on their theorized influence on adolescent-girl outcomes, while also being directly linked to the intervention activities and curriculum topics. The following eight mediating indicators reflecting girls’ social, health and economic assets were evaluated among all girls: (1) **Social Assets** – a) self-efficacy, measured as a score with 0–10 range based on the Generalized Self-Efficacy Scale [17]; b) whether the girl had a safe space in the community to meet with friends, c) positive gender attitudes, with questions based on measures developed in previous studies [11, 18]. This indicator ranged from 0 to 7; and d) non-acceptability of intimate partner violence (IPV), based on Demographic and Health Survey (DHS) questions, measured as a binary variable that is equal to 1 if the respondent did not agree that a husband is justified in hitting his wife for any of five specific reasons; (2) **Economic Assets** – a) financial literacy, measured based on previous studies [11, 18, 19] as a score ranging from 0 to 9; b) a dichotomous variable of whether the girl saved any money in the past year; and (3) **Health Assets** - a) knowledge of fertile period and contraceptive methods based on DHS questions, measured as a score with 0–11 range, and b) HIV/AIDS knowledge based on DHS-related questions, measured as a score with 0–11 range. The following two mediating indicators reflecting sexual behavior were evaluated among girls aged 15 years and older who had initiated sex: (1) used condom at first sex; and (2) reported having had transactional sex. The mediating indicators, while reflecting the original intent of the proposed mediating outcomes in the initial study protocol submitted for ethical review, were revised after baseline to capture the most valid and reliable measures still in line with the theoretical framework (published elsewhere [1]).

Two educational outcomes were evaluated among all girls: (1) completed the last grade of primary school (grade 7); and (2) completed grade 9. The following four fertility longer-term outcomes were evaluated among girls aged 15 years and older: (1) ever had sex; (2) ever been pregnant; (3) ever given birth; and (4) ever been married. All of these were identified as primary long-term outcomes in the initial study protocol submitted for ethical review.

### Analytical sample

This paper uses the sample of girls interviewed at baseline, Round 3 (end of the intervention) and Round 5 (2 years after the end of the intervention). Baseline data were collected between July 2013 and February 2014. Round 3 was conducted between July 2015 and January 2016 among all girls interviewed at baseline. Round 5 was conducted between July and December 2017. Due to budgetary constraints, a sub-sample of girls interviewed at Round 3 was randomly selected for participation in Round 5.

### Statistical analysis

To assess attrition bias, a probit regression where the outcome was equal to one if the respondent was excluded from the analytical sample was estimated. Covariates included the respondent’s study arm, the following **socio-demographic characteristics** measured at baseline: age; attended school in the current year; grade attainment; literate, defined as reading out loud a full sentence in English or a local language; mother and father alive and co-residence status; mother’s and father’s completion of primary school; household wealth quintiles, derived from a wealth index estimated using principal-components analysis; vulnerability quintiles derived from the vulnerability score estimated to target girls for invitation to AGEP; and dummy variables for program sites. To evaluate baseline balance across the intervention and control arms among girls in the analytical sample, means, 95% confidence intervals and Pearson chi-square tests for categorical variables and linear regression for continuous variables, accounting for sample clustering at the CSA level, were estimated for the set of socio-demographic characteristics listed above. Means and 95% confidence intervals, accounting for clustering at the CSA level, were also estimated for mediating indicators and **outcome variables** measured at baseline and at Rounds 3 and 5 to explore change across time.

The primary impact analysis was based upon an “intent-to-treat” (ITT) approach for outcomes measured at baseline and Rounds 3 and 5. The ITT parameter of impact was defined as whether a girl was invited to participate in AGEP, regardless of her actual participation in the program. Linear regressions with girl-level fixed effects were estimated. All regressions were estimated with robust standard errors adjusted for clustering at the CSA level, and all models controlled for girl’s age. Difference-in-differences (DID) were estimated, comparing the change between Round 1 and Rounds 3 and 5 for intervention versus control arms.

To account for various levels of participation in AGEP observed across girls—due to girls not signing up for the program, drop-out or absences—a secondary analysis was conducted to obtain “treatment-on-the-treated” (TOT) estimates from two-stage least squares, instrumental variable (IV) regressions. The TOT estimates relied on instrumentation to account for unobservable differences that may have contributed to differential program participation.^5^ These unobservable factors may be correlated with the adolescent girl outcomes and would lead to biased estimates of impact if not statistically accounted for in the analysis. The first stage of the estimations predicted program participation, with the indicator of treatment defined as attendance to at least 52 meetings (half of the total girls group meetings). The instrumental variable used in the first stage was the randomized invitation to participate in AGEP. The validity of the instrumental variable was assessed with F-tests on excluded instruments, the Wald F-statistic and the Hansen statistic for overidentification. DID coefficients were estimated in the second stage of the regressions. All regressions were estimated with robust standard errors adjusted for clustering at the CSA level, and all models controlled for girl’s age.

After the primary (ITT) and secondary analyses (TOT) were conducted, we explored whether the program had differential impacts at Round 5 across sub-groups of girls. To this end, we re-estimated the ITT regressions including interaction terms between the treatment indicators and the following characteristics: urban vs rural, older (aged 15–19 at baseline) vs younger (aged 10–14 at baseline), highest vulnerability quintile vs lower vulnerability quintiles, and poorest household wealth quintile vs less poor household wealth quintiles.^6^ For example, to test whether the intervention had higher impact for girls who were in urban sites at baseline, the ITT regressions were re-estimated including interactions of the DID term with a variable indicating whether the girl was living in an urban site at baseline or not. The coefficient of this interaction indicated whether the impact of the intervention differed by baseline location. A similar approach was used to test the effects of age, levels of vulnerability and household wealth at baseline. All regressions here and above were estimated in Stata® 15.1.

## Results

**Fig. 1:**
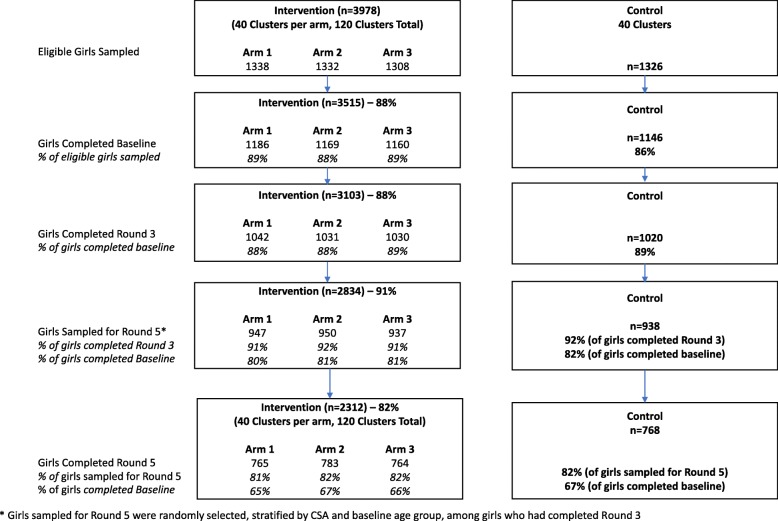
Analytical sample flow

Figure 1 shows the analytical sample flow by study arms. At baseline, 4661 never-married girls ages 10–19 were successfully interviewed, representing 88% of eligible girls drawn in the sampling frame.^7^ In Round 3, 88% of girls in the baseline sample were interviewed. In Round 5, 82% of girls in the Round 5 sampling frame (or 66% of the baseline sample) were interviewed. No differential attrition between intervention and control arms was observed. Girls interviewed at Round 5 constituted the analytical sample used in this paper, as all girls interviewed at Round 5 had been interviewed both at baseline and at Round 3. Results from the probit regression estimated to compare girls who were lost to follow-up to girls included in the analytical sample are shown in Additional file 2. Study arms were not associated with being excluded from the analytical sample. Girls excluded from the analytical sample were, at baseline, statistically significantly older and more likely to not co-reside with their mothers, they were also less likely to be in school and had lower grade attainment at baseline than girls included in the analytical sample.

Table 1 shows descriptive statistics among girls in the analytical sample for selected baseline socio-demographic characteristics by intervention and control arms. As the analytical sample excluded girls who were part of the baseline sample but were lost to follow-up, tests of differences across arms were performed to assess whether attrition led to baseline unbalances across arms. As seen in the table, none of the F statistics were significant at the 0.05 level, thus while attrition was not random (as discussed in the previous paragraph), it did not affect the baseline balance.

**Table 1:** Baseline socio-demographic characteristics of analytical sample, by intervention and control arms

	Intervention arms									Control arm			
	Arm 1			Arm 2			Arm 3						
	Mean	95% CI^a^		Mean	95% CI^a^		Mean	95% CI^a^		Mean	95% CI^a^		F-stat^b^
Age	14.3	14.1	14.4	14.2	14.0	14.3	14.2	14.1	14.4	14.2	14.1	14.4	0.22
Had attended current school year	81%	78%	84%	82%	78%	85%	82%	79%	85%	81%	78%	84%	0.04
Highest grade completed	5.5	5.2	5.7	5.4	5.2	5.7	5.6	5.4	5.8	5.6	5.4	5.9	0.62
Literate	43%	39%	48%	43%	38%	47%	46%	42%	51%	48%	44%	53%	1.35
Mother’s living and co-residence status													
Co-resident with girl	72%	69%	76%	68%	65%	72%	66%	62%	71%	71%	66%	75%	
Alive but not co-resident with girl	18%	16%	21%	18%	15%	21%	21%	17%	25%	17%	14%	21%	
Not alive	9%	7%	12%	14%	11%	16%	12%	10%	15%	12%	10%	14%	1.50
Father’s living and co-residence status													
Co-resident with girl	52%	48%	56%	48%	45%	52%	48%	44%	52%	51%	45%	56%	
Alive but not co-resident with girl	27%	23%	30%	27%	24%	31%	29%	25%	33%	28%	24%	32%	
Not alive	22%	18%	25%	24%	21%	27%	23%	20%	26%	21%	18%	25%	0.52
Mother completed primary school	41%	38%	45%	47%	42%	51%	49%	46%	53%	46%	42%	50%	2.62^†^
Father completed primary school	49%	45%	52%	53%	50%	57%	52%	48%	57%	54%	49%	58%	1.33
Household wealth quintiles													
1 Poorest	21%	17%	25%	18%	14%	22%	19%	15%	23%	20%	16%	24%	
2	20%	16%	24%	19%	15%	22%	17%	14%	20%	20%	16%	23%	
3	22%	19%	26%	20%	17%	23%	20%	16%	23%	18%	15%	21%	
4	19%	16%	23%	21%	18%	24%	21%	18%	24%	23%	19%	27%	
5 Wealthiest	17%	12%	21%	22%	17%	27%	24%	17%	30%	20%	15%	25%	0.94
Vulnerability quintiles													
1 Lowest vulnerability	25%	22%	28%	22%	19%	25%	26%	22%	29%	25%	21%	29%	
2	19%	17%	22%	20%	17%	23%	20%	17%	23%	20%	17%	22%	
3	21%	18%	23%	22%	19%	25%	20%	18%	23%	23%	20%	26%	
4	18%	16%	21%	17%	14%	20%	18%	15%	21%	16%	14%	19%	
5 Highest vulnerability	17%	14%	20%	19%	15%	22%	16%	13%	19%	16%	13%	19%	0.54
Number of girls in analytical sample	765			783			764			768			

^a^ Confidence intervals (CI) were estimated accounting for clustering at the CSA level

^b^ F-statistic from Pearson design-based tests for categorical variables and from linear regressions for continuous variables, accounting for clustering at the CSA level

*** *p* < 0.001, ** *p* < 0.01, * *p* < 0.05, † *p* < 0.1

Table 2 provides the baseline and Rounds 3 and 5 means, by study arms, among the analytical sample for the primary indicators of interest across the three domains of empowerment addressed in AGEP, social assets, economic assets and health assets, as well as indicators of sexual behavior among respondents ages 15 and older who had ever had sex. In the domain of social assets, at baseline, respondents showed middling levels of self-efficacy, with an average score of 6 to 6.1, within a potential range of 0 to 10. Self-efficacy levels increased over time as respondents in the intervention arms scored 7.4 to 7.7 out of 10 and respondents in the control arm scored 7.3 at Round 5. A high percentage of girls (58 to 62%) did not have access to a safe place in the community to meet with friends at baseline, which contributed towards their social isolation. The percentage of respondents who reported no access to a safe space decreased over time from 45 to 48% of respondents in the intervention arms and 48% of respondents in the control arm at Round 5. While girls on average held positive gender norms (scoring 4.9 to 5.1 out of 7 at baseline), generally there was also a high acceptance of intimate partner violence (IPV) as 60 to 63% agreed at baseline that hitting/striking a spouse was acceptable in certain circumstances. No change was observed over time in measured respondents’ gender norms, but acceptance of IPV appeared to increase as girls aged. For measured economic assets, at baseline, respondents had an average score of 5.1 to 5.3 out of 9 in financial literacy, and only 14 to 16% had saved money in the past year. Financial literacy increased over time as respondents in the intervention arms scored 6.2 to 6.4 out of 9 and respondents in the control arm scored 6.2 at Round 5, and the percentage of respondents who had saved money in the past year more than doubled (39 to 41% of respondents in the intervention arms and 33% of respondents in the control arm at Round 5).

**Table 2:** Mediating indicators and longer term outcomes among analytical sample, by intervention and control arms and by survey rounds

	Intervention arms									Control arm		
	Arm 1			Arm 2			Arm 3					
	Mean	95% CI^a^		Mean	95% CI^a^		Mean	95% CI^a^		Mean	95% CI^a^	
Social assets												
Self-efficacy score [0–10]												
Baseline	6.0	5.8	6.3	6.1	5.9	6.4	6.1	5.9	6.2	6.1	5.9	6.3
Round 3	7.0	6.8	7.2	7.2	7.1	7.4	7.2	7.0	7.3	7.0	6.8	7.2
Round 5	7.4	7.3	7.6	7.6	7.4	7.7	7.7	7.5	7.8	7.3	7.1	7.5
Had a safe space in community to meet with friends												
Baseline	38%	34%	42%	42%	39%	45%	42%	39%	46%	40%	36%	44%
Round 3	46%	41%	51%	49%	45%	52%	47%	43%	52%	38%	34%	43%
Round 5	55%	51%	58%	54%	50%	58%	52%	48%	56%	52%	48%	56%
Positive gender attitudes score [0–7]												
Baseline	4.9	4.8	5.1	4.9	4.8	5.0	5.0	4.9	5.1	5.0	4.8	5.1
Round 3	4.8	4.7	4.9	4.8	4.7	5.0	5.0	4.9	5.1	4.9	4.8	5.0
Round 5	4.9	4.7	5.0	5.0	4.8	5.1	5.0	4.8	5.1	5.0	4.8	5.2
Non-acceptability of IPV												
Baseline	40%	35%	44%	37%	33%	41%	38%	34%	42%	40%	36%	44%
Round 3	33%	30%	37%	37%	32%	41%	42%	37%	46%	40%	36%	44%
Round 5	34%	29%	38%	37%	33%	41%	36%	32%	41%	38%	33%	42%
Economic assets												
Financial literacy score [0–9]												
Baseline	5.1	5.0	5.3	5.1	4.9	5.4	5.2	5.0	5.4	5.3	5.1	5.5
Round 3	5.9	5.7	6.1	6.0	5.8	6.1	6.1	6.0	6.2	5.9	5.7	6.0
Round 5	6.2	6.0	6.4	6.2	6.0	6.3	6.4	6.2	6.5	6.2	6.0	6.3
Saved money in the past year												
Baseline	16%	13%	19%	15%	11%	18%	14%	11%	17%	15%	11%	18%
Round 3	30%	26%	34%	33%	29%	37%	35%	31%	38%	26%	22%	29%
Round 5	39%	35%	43%	40%	37%	44%	41%	38%	44%	33%	30%	36%
Health assets												
Fertile period and contraceptive methods knowledge score [0–11]												
Baseline	1.6	1.4	1.7	1.7	1.5	1.8	1.7	1.6	1.8	1.7	1.6	1.9
Round 3	2.7	2.5	2.8	2.8	2.6	3.0	2.7	2.6	2.9	2.5	2.4	2.7
Round 5	3.6	3.4	3.8	3.6	3.4	3.8	3.8	3.6	4.0	3.5	3.3	3.6
HIV knowledge score [0–11]												
Baseline	5.7	5.4	6.0	5.9	5.6	6.2	6.1	5.7	6.4	5.9	5.6	6.2
Round 3	8.1	7.8	8.3	8.3	8.1	8.5	8.2	8.0	8.5	8.1	7.8	8.3
Round 5	8.9	8.7	9.0	9.0	8.9	9.2	9.1	8.9	9.2	8.9	8.8	9.0
Sexual behavior among girls ages 15 and older who had ever had sex												
Used condom at first sex^b^												
Baseline	41%	32%	50%	46%	38%	55%	34%	27%	40%	39%	30%	48%
Round 3	42%	36%	48%	44%	37%	50%	40%	35%	44%	38%	32%	44%
Round 5	42%	37%	48%	43%	38%	48%	42%	38%	46%	41%	37%	46%
Agreed to having had transactional sex^c^												
Baseline	56%	47%	64%	59%	51%	67%	58%	50%	66%	48%	39%	56%
Round 3	45%	40%	49%	44%	37%	51%	47%	41%	53%	49%	44%	55%
Round 5	40%	35%	45%	36%	31%	42%	40%	35%	45%	39%	33%	46%
Education outcomes												
Completed grade 7												
Baseline	37%	33%	41%	37%	33%	41%	40%	36%	44%	41%	37%	45%
Round 3	52%	48%	57%	55%	50%	60%	55%	51%	59%	57%	53%	62%
Round 5	68%	63%	72%	68%	63%	73%	70%	65%	74%	69%	65%	73%
Completed grade 9												
Baseline	12%	9%	15%	11%	8%	14%	11%	7%	14%	12%	9%	16%
Round 3	24%	20%	28%	24%	20%	28%	22%	18%	26%	25%	21%	28%
Round 5	35%	30%	39%	34%	29%	39%	34%	29%	38%	37%	32%	41%
Fertility outcomes among girls ages 15 and older^d^												
Ever married												
Baseline	0%			0%			0%			0%		
Round 3	20%	17%	23%	19%	15%	23%	16%	13%	19%	16%	12%	20%
Round 5	28%	25%	31%	29%	25%	33%	29%	25%	33%	26%	21%	31%
Ever had sex												
Baseline	40%	34%	46%	39%	34%	44%	44%	38%	49%	42%	37%	47%
Round 3	65%	60%	70%	65%	60%	69%	67%	63%	72%	61%	56%	66%
Round 5	74%	71%	77%	75%	70%	79%	77%	73%	80%	69%	64%	73%
Ever pregnant												
Baseline	15%	11%	20%	14%	11%	18%	16%	13%	20%	17%	13%	20%
Round 3	34%	29%	39%	33%	29%	38%	34%	30%	39%	34%	29%	39%
Round 5	46%	42%	50%	46%	42%	51%	49%	46%	53%	44%	39%	49%
Ever given birth												
Baseline	10%	6%	14%	9%	6%	12%	11%	7%	14%	13%	10%	15%
Round 3	25%	21%	30%	25%	21%	28%	27%	23%	32%	27%	22%	32%
Round 5	38%	34%	42%	38%	34%	43%	40%	36%	43%	38%	34%	42%

^a^ Confidence intervals (CI) were estimated accounting for clustering at the CSA level

^b^ Number of girls age 15 and older who had ever had sex and for whom data on condom use at first sex is available: 535 at baseline; 1131 at round 3; and 1720 at round 5

^c^ Number of girls age 15 and older who had ever had sex and for whom data on transactional sex is available: 534 at baseline; 1142 at round 3; and 1732 at round 5

^d^ Number of girls age 15 and older: 1500 at baseline; 2150 at round 3; and 2669 at round 5

In the domain of health assets, while knowledge of the fertile period and contraceptive methods was low at baseline (1.6 to 1.7 out of 11), levels of HIV knowledge were higher (5.7 to 6.1 out of 11). On average, scores for both indicators increased over time, but knowledge of the fertile period and contraceptive methods remained low (3.6 to 3.8 out of 11 among respondents in the intervention arms and 3.5 among respondents in the control arm at Round 5), while HIV knowledge score reached 8.9 to 9.1 out of 11 among respondents in the intervention arms and 8.9 among respondents in the control arm at Round 5. Among girls 15 years and older who had reported having had sex at baseline, 34 to 46% reported having used a condom at first sex, and 48 to 59% agreed to having had transactional sex. Over time, as a larger group of girls in the analytical sample became ages 15 and older and experienced sex, the percentage of girls who had used a condom at first sex was only slightly higher (41 to 43% at Round 5) than at baseline, but the percentage of girls who agreed to having had transactional sex became considerably lower (36 to 40% at Round 5).

Table 2 also provides the prevalence of the longer-term educational and fertility outcomes of interest at baseline and Rounds 3 and 5. At baseline, 37 to 41% of respondents had completed grade 7, and 11 to 12% had completed grade 9. At Round 5, these figures were 68 to 70% and 34 to 37%, respectively. Among girls 15 years and older: at baseline, 39 to 44% had ever had sex, 14 to 17% had ever been pregnant and 9 to 13% had given birth. The baseline sample purposely included only never-married girls. Over time, as girls aged and a larger group of girls became ages 15 and older, the prevalence of all four outcomes increased considerably. Among girls 15 years and older at Round 5, 26 to 29% had ever been married, 69 to 77% had ever had sex, 44 to 49% had ever been pregnant and 38 to 40% had given birth.

The **ITT estimates** for each of the outcomes of interest are shown in Table 3. The three intervention arms are combined and compared against the control arm, as estimates by arms showed few substantial differences across intervention arms on outcomes (results shown in Additional file 3).^8^ The left-hand panel of the table shows the coefficients representing the estimated DID between baseline and R3, and the right-hand panel shows the coefficients representing the estimated DID between baseline and R5. The table also shows 95% confidence intervals (CIs) for the estimated DID. Of the eight asset empowerment indicators, the program improved outcomes relative to the control at Round 3 across four indicators: 1) having a safe space in the community to meet with friends increased 8.2 percentage points (95% CI 1.7–14.6), 2) financial literacy score saw a modest increase of 0.24 points out of 9 (95% CI 0.01–0.48), whether girls had saved money in the past year increased 6.7 percentage points (95% CI 1.8–11.5) and sexual and reproductive health knowledge also saw a modest increase of 0.29 points out of 11 (95% CI 0.10–0.48). At Round 5, improved outcomes relative to the control are still observed in saving money (DID coef 6.7, 95% CI 2–11.3) and sexual and reproductive health knowledge (DID coef 0.27, 95% CI 0.06–0.48), and positive but modest program impacts emerged in self-efficacy with a 0.31 higher score out of 10 (95% CI 0.02–0.6). Program impacts were not observed for girls’ HIV knowledge and gender norms.

**Table 3:** Estimated difference-in-differences (DID) for intent-to-treat (ITT), results from linear regressions with girl-level fixed effects

	Round 3			Round 5		
	DID coef	95% CI		DID coef	95% CI	
Social assets						
Self-efficacy score [0–10]	0.100	−0.165	0.366	0.310*	0.024	0.595
Had a safe space in community to meet with friends	0.082*	0.017	0.146	0.012	−0.048	0.071
Positive gender attitudes score [0–7]	−0.010	−0.196	0.177	0.003	−0.229	0.234
Non-acceptability of IPV	−0.007	−0.067	0.053	0.007	−0.059	0.073
Economic assets						
Financial literacy score [0–9]	0.243*	0.010	0.476	0.209	−0.056	0.474
Saved money in the past year	0.067**	0.018	0.115	0.067**	0.020	0.113
Health assets						
Fertile period and contraceptive methods knowledge score [0–11]	0.289**	0.095	0.483	0.268*	0.056	0.481
HIV knowledge score [0–11]	0.107	−0.263	0.477	0.107	− 0.270	0.485
Sexual behavior among girls ages 15 and older who had ever had sex						
Used condom at first sex^a^	0.047	−0.042	0.136	0.010	−0.053	0.073
Agreed to having had transactional sex	−0.119*	−0.234	− 0.005	−0.118*	− 0.219	− 0.017
Education outcomes						
Completed grade 7	−0.004	− 0.041	0.033	0.017	−0.024	0.058
Completed grade 9	−0.002	− 0.038	0.034	− 0.015	− 0.062	0.033
Fertility outcomes among girls ages 15 and older						
Ever married	0.011	−0.046	0.068	0.023	−0.057	0.103
Ever had sex	0.043	−0.011	0.098	0.069*	0.010	0.127
Ever pregnant	−0.013	−0.078	0.052	0.033	−0.035	0.102
Ever given birth	0.006	−0.059	0.071	0.028	−0.039	0.095

All models adjust for age. Robust standard errors adjusted for clusters at the CSA level

*** *p* < 0.001, ** *p* < 0.01, * *p* < 0.05, † *p* < 0.1

^a^ Estimated as simple differences at each round between intervention and control arms excluding girls who had ever had sex at baseline and adjusting for age and study site

For the sexual behavior indicators, the ITT estimates indicated that that while there was no impact on condom use at first sex, the program had a positive impact in decreasing the prevalence of transactional sex relative to the control by 11.9 percentage points at Round 3 (95% CI -23.4–-0.5) and by 11.8 percentage points at Round 5 (95% CI -21.9–-1.7), suggesting meaningfully lower exposure to adverse sexual reproductive health risks.

No positive program impacts were observed, neither at the end of the intervention (Round 3), nor two years after the end of the intervention (Round 5), for the primary education and fertility outcomes.

The **TOT estimates** are provided in Table 4. Estimates by intervention arms are presented in Additional file 4. The left-hand panel of the table shows the coefficients representing the estimated DID between baseline and R3, and the right-hand panel shows the coefficients representing the estimated DID between baseline and R5. The table also shows 95% confidence intervals for the estimated DID. The estimates from the two-stage, instrumental-variables regressions indicate what the “undiluted” effect of the program impact is assuming that only girls who participated in at least 52 sessions were affected by the program. Across the indicators, the TOT estimates were approximately three times as large as the ITT estimates.

**Table 4:** Estimated difference-in-differences (DID) for treatment-on-the-treated (TOT), results from two-stage least squares IV regressions with girl-level fixed-effects

	Round 3			Round 5		
	DID coef	95% CI		DID coef	95% CI	
Social assets						
Self-efficacy score [0–10]	0.290	−0.470	1.051	0.892*	0.076	1.708
Had a safe space in community to meet with friends	0.236*	0.049	0.423	0.033	−0.138	0.204
Positive gender attitudes score [0–7]	− 0.028	−0.561	0.504	0.008	−0.651	0.666
Non-acceptability of IPV	−0.019	−0.190	0.152	0.020	−0.167	0.207
Economic assets						
Financial literacy score [0–9]	0.701*	0.032	1.369	0.601	−0.153	1.354
Saved money in the past year	0.193**	0.055	0.331	0.191**	0.058	0.325
Health assets						
Fertile period and contraceptive methods knowledge score [0–11]	0.831**	0.275	1.388	0.770*	0.163	1.377
HIV knowledge score [0–11]	0.309	−0.746	1.364	0.309	− 0.762	1.379
Sexual behavior among girls ages 15 and older who had ever had sex						
Used condom at first sex^a^	0.187	−0.170	0.544	0.032	−0.171	0.235
Agreed to having had transactional sex	−0.524*	−1.032	−0.016	− 0.515*	−0.967	− 0.063
Education outcomes						
Completed grade 7	−0.012	−0.118	0.094	0.050	−0.067	0.166
Completed grade 9	−0.005	−0.108	0.098	−0.042	− 0.178	0.094
Fertility outcomes among girls ages 15 and older						
Ever married	0.041	−0.166	0.249	0.083	−0.200	0.366
Ever had sex	0.161	−0.034	0.355	0.246*	0.036	0.455
Ever pregnant	−0.042	− 0.274	0.190	0.115	− 0.128	0.357
Ever given birth	0.024	−0.207	0.256	0.098	−0.140	0.337

All models adjust for age. Robust standard errors adjusted for clusters at the CSA level

*** *p* < 0.001, ** *p* < 0.01, * *p* < 0.05, † *p* < 0.1

^a^ Estimated as simple differences at each round between intervention and control arms excluding girls who had ever had sex at baseline and adjusting for age and study site

To assess the potential heterogeneity of program impact, additional estimation results are presented in Table 5. The table provides ITT assessments of impact on sub-groups that were hypothesized to potentially benefit differentially from the program: urban vs rural sites; older (aged 15–19 at baseline) vs younger (aged 10–14 at baseline); poorest household wealth quintile vs less-poor household wealth quintiles; and, highest-vulnerability quintile vs lower-vulnerability quintiles. The results in Table 5 indicated that there were few meaningful differences among the mediating indicators of interest for sub-groups: relative to the younger cohort of girls, the impact on self-efficacy was 0.49 points out of 10 higher for older girls (95% CI − 0.02–1) and non-acceptability of IPV was 11.7 percentage points higher for older girls (95% CI -0.5–23.9). The results for the longer-term fertility outcomes showed, however, girls in the intervention arms in urban sites were 13.5 percentage points (95% CI 3.3–23.7) more likely to have initiated sex than girls in the rural sites. Further, the most vulnerable girls in the intervention arms were 16.2 percentage points more likely to be married (95% CI 0.8–31.7), 28 percentage points more likely to become pregnant (95% CI 14.1–41.8) and 21.3 percentage points more likely to give birth (95% CI 7.1–35.4). These results suggest that the lack of impact (statistical and substantive) in the ITT estimates in Table 3 is not likely masking underlying significant impacts for certain sub-groups, while the apparent negative impact on sex initiation in Table 3 seems driven by girls in the urban sites.

**Table 5:** Estimated DID interaction effects between baseline and Round 5 for intent-to-treat (ITT), results from linear regressions with girl-level fixed effects

	Interaction with urban site (vs rural site)			Interaction with older cohort (vs younger cohort)			Interaction with poorest HHs (vs. less poor HHs)			Interaction with highest vulnerability (vs lower vulnerability)		
	Coef	95% CI		Coef	95% CI		Coef	95% CI		Coef	95% CI	
Social assets												
Self-efficacy score [0–10]	−0.133	− 0.710	0.444	0.491^†^	−0.020	1.002	−0.114	− 0.787	0.559	0.255	−0.356	0.867
Had a safe space in community to meet with friends	0.011	−0.108	0.130	0.033	−0.103	0.168	0.031	−0.120	0.181	−0.068	− 0.257	0.122
Positive gender attitudes score [0–7]	− 0.146	− 0.601	0.310	− 0.062	− 0.292	0.169	− 0.052	− 0.433	0.329	0.152	−0.272	0.576
Non-acceptability of IPV	−0.090	− 0.215	0.035	0.117^†^	− 0.005	0.239	0.002	− 0.146	0.150	0.074	− 0.064	0.212
Economic assets												
Financial literacy score [0–9]	− 0.143	− 0.675	0.389	0.135	−0.224	0.494	−0.218	− 0.744	0.309	0.082	−0.432	0.596
Saved money in the past year	0.002	−0.092	0.095	0.037	−0.049	0.124	−0.002	−0.109	0.104	0.048	−0.067	0.163
Health assets												
Fertile period and contraceptive methods knowledge score [0–11]	− 0.035	−0.460	0.390	0.301	−0.111	0.713	−0.170	−0.706	0.367	0.078	−0.460	0.615
HIV knowledge score [0–11]	−0.020	−0.775	0.736	−0.462	−1.086	0.163	−0.382	−1.128	0.364	−0.089	− 0.882	0.703
Sexual behavior among girls ages 15 and older who had ever had sex												
Used condom at first sex^a^	0.023	−0.104	0.150				0.072	−0.083	0.228	−0.127	−0.320	0.066
Agreed to having had transactional sex	−0.043	−0.257	0.170				−0.068	−0.432	0.295	−0.192	− 0.511	0.126
Education outcomes												
Completed grade 7	0.001	−0.080	0.083	−0.023	−0.101	0.056	0.005	−0.085	0.096	−0.031	−0.133	0.072
Completed grade 9	0.036	−0.058	0.130	0.062	−0.012	0.136	0.037	−0.054	0.129	−0.020	−0.096	0.055
Fertility outcomes among girls ages 15 and older												
Ever married	−0.010	−0.169	0.150				0.110	−0.046	0.266	0.162*	0.008	0.317
Ever had sex	0.135*	0.033	0.237				0.024	−0.120	0.168	0.030	−0.127	0.187
Ever pregnant	0.052	−0.081	0.185				0.106	−0.064	0.275	0.280***	0.141	0.418
Ever given birth	0.052	−0.079	0.183				0.133	−0.041	0.307	0.213**	0.071	0.354

All models adjust for age. Robust standard errors adjusted for clusters at the CSA level

*** *p* < 0.001, ** *p* < 0.01, * *p* < 0.05, † *p* < 0.1

^a^ Estimated as simple differences at Round 5 between intervention and control arms excluding girls who had ever had sex at baseline and adjusting for age and study site

## Discussion

The results presented in this paper assessing the impact of a girls’ empowerment program add to the literature evaluating interventions that focus on building girls’ assets as means to overcome their vulnerability. AGEP was designed to improve shorter-term outcomes as captured by social, health and economic assets, with the goal of improving longer-term educational and fertility outcomes such as delayed sexual debut, marriage and pregnancy. Recent systematic reviews of the published and grey literature on interventions to improve outcomes of unintended pregnancy, STIs and child marriage among adolescents have shown that a diversity of interventions have effects, yet there is no one intervention that works in all contexts and for all outcomes [20–22]. For example, a combined economic strengthening and sexual and reproductive health education intervention for adolescent girls increased income generation, reduced pregnancy and delayed marriage in Uganda [14], yet when replicated by the same organizations in Tanzania, the interventions had no effect [23]. In the case of AGEP in Zambia, the hypothesis was not confirmed, and although there was sustained change on a limited number of assets, namely SRH knowledge, self-efficacy and savings, the intervention did not lead to a combined set of social, health and economic assets. In addition, the shorter-term changes that did occur did not result in longer-term impacts on education or fertility for vulnerable girls in the Zambian context.

Several factors must be considered in understanding why the intervention did not result in the hypothesized changes. One factor is the low participation, as only 30% of the sample participated in half or more of the program sessions, and 25% did not participate at all. While the TOT analysis did not show meaningful change for those who did participate, understanding who attended AGEP sessions is one component of understanding the effects of the program, as well as potential gaps in content. Given that AGEP purposefully targeted the most vulnerable girls in each community, it is likely that they face social and economic barriers at the individual, household and community levels that prevent both participation and effectiveness of the program components. It is possible that a program focused on girls’ asset building alone, even if it combined health and economic components, was not meaningful enough for girls and their families to lead to participation. Potentially, girls from the most disadvantaged households need additional interventions that address the household economic status, which may prevent girls from participating in the first place. It is also possible that without addressing the economic constraints at the household level, even participation in a girls’ empowerment program alone is not enough to impact longer-term outcomes such as educational attainment or timing of pregnancy. More research needs to be done to understand what will overcome the barriers to participation for the most disadvantaged, even if the findings suggest that more complex, and likely costly, interventions are necessary. One particularly promising intervention approach might be the combination of targeted cash transfers and direct girls’ empowerment programming [24], as it simultaneously can address household poverty, as well as build individual assets for girls.

A second factor is the social and cultural context in which this intervention took place. With 60% of girls agreeing that intimate partner violence is acceptable, two-thirds having experienced physical violence and half having experienced sexual violence (defined as being forced into sexual intercourse or other sexual acts), it is likely that an intervention targeting only asset building for girls is not sufficient to change attitudes around gender roles or acceptability of violence. Taking a socio-ecological approach [25] in the future may lead to different results. Future programs in this context need to address norms at the household and community level, in addition to direct interventions with girls. This is especially relevant in thinking about the beliefs held by the mentors – the women facilitating the weekly group sessions – and how the social norms around gender to which they ascribe mediate the translation of program content to the participants in their groups. This is reinforced by looking at the type of outcomes for which there was positive change – self-efficacy, sexual and reproductive health knowledge, savings behavior and transactional sex – these are potentially outcomes over which an adolescent may exercise more control. Other outcomes – gender norms, recent condom use, school completion, timing of marriage – involve external factors (e.g. parents and sexual partners), and therefore may be harder to change with an intervention that focuses directly on girls.

There are several limitations to the study and interpretation of its results. The first is this is one study done in one cultural context, and the ability to generalize to other settings may be limited. Of course, this is a limitation of all studies. The second is the greatly reduced sample for Round 5, which likely reduced precision and introduced selection bias into the analytical sample vis-à-vis age, mother’s co-residence and educational attainment. This could impact the validity of our findings. Third, the trial was not registered prospectively, however the design was not changed according to the protocol submitted for ethical approval. Finally, we did not correct for the testing of multiple hypotheses. However, given the lack of positive results, we do not think this would influence our findings.

## Conclusions

Overall, the results of the AGEP trial provide an important contribution to the field of adolescent programming and understanding what combinations of interventions work and do not work, in different contexts. These findings suggest that when addressing long-term educational and fertility outcomes among very vulnerable adolescent girls, interventions that only target the girl herself may not be sufficient.

## Supplementary information

**Additional file 1.** Mediating indicators and longer-term outcomes.

**Additional file 2.** Results from a probit regression for attrition.

**Additional file 3.** Estimated difference-in-differences (DID) for intent-to-treat (ITT) by intervention arm, results from linear regressions with girl-level fixed effects.

**Additional file 4.** Estimated difference-in-differences (DID) for treatment-on-the-treated (TOT) by intervention arms, results from two-stage least squares IV regressions with girl-level fixed-effects.

## Data Availability

The de-identified datasets from the first four rounds of data collection, which were generated and analyzed during the current study, are available in the Adolescent Data Hub repository, 10.7910/DVN/CFIUC6. The final round of data collection will be available at that location by mid-2020; until that time it is available from the corresponding author on reasonable request.

## Abbreviations

AGEP: Adolescent Girls Empowerment Program
DHS: Demographic and Health Survey
DID: Difference in Difference
GSE: Generalized Self-Efficacy
HIV: Human Immuno-deficiency Virus
HSV-2: Herpes Simplex Virus – Type 2
IPV: Intimate Partner Violence
ITT: Intent to Treat
ODK: Open Data Kit
OLS: Ordinary Least Squares
SRH: Sexual and Reproductive Health
TOT: Treatment on the Treated

## Footnotes

1 Adolescence in this paper is defined using the World Health Organization definition of 10–19 years old - http://www.who.int/topics/adolescent_health/en/

2 A CSA contains a collection of adjacent standard enumeration areas (SEA) that range in number from two to eight per CSA. SEAs are geographical areas that contain approximately 100 households in rural areas and 150 households in urban areas.

3 In some clusters there were fewer than 17 girls aged 10–14 and/or 23 girls aged 15–19, in which case all girls were selected for the research sample. This led to an initial sampling frame of 5304.

4 While the study was retrospectively registered in the ISRCTN Registry, the study protocol was submitted for ethical review prior to any data being collected or randomization assignments made. There were no changes to the initial study design, intervention design or analysis plan.

5 Analysis of program uptake and participation showed that 26% of girls invited never attended, 44% of girls attended 52 sessions or less (representing half of all potential sessions one could attend) and 30% attended more than 52 sessions. The mean number of sessions attended (32.8) was balanced across program arms, therefore program arm assignment or the added components of the intervention did not affect program participation. There was however differential program uptake with girls who were younger, living in rural areas, in school, with higher numeracy skills, and the biological daughter of the head of household being the most likely to participate in a greater number of sessions.

6 Secondary analysis of results by urban v. rural and older v. younger stratification was included in the original protocol and sample size calculations took these subgroups into account. Additional subgroup analysis on SES and vulnerability score were added at the time of analysis to determine if the program effects on particularly vulnerable subgroups were being washed out (or were washing out) effects on relatively less vulnerable sub-groups.

7 14% of girls in the research sampling frame were found not to be eligible as they had relocated, married, or were outside of the age range.

8 The analysis plan in the protocol described estimates by arms (Appendix 3), however after initial estimates showed little difference by arm, the authors decided to present the combined estimates.
