# Supplementary material for: The impact of the Adolescent Girls Empowerment Program (AGEP) on short and long term social, economic, education and fertility outcomes: a cluster randomized controlled trial in Zambia
Source: BMC Public Health. 2020 Mar 17;20:349. doi: 10.1186/s12889-020-08468-0 (PMC7079524; doi:10.1186/s12889-020-08468-0)
Supplement: Supplementary file 1 — Additional file 1. Mediating indicators and longer-term outcomes. [file 12889_2020_8468_MOESM1_ESM.docx]

**Appendix 1: Mediating indicators and longer-term outcomes**

|  | Definition and survey items | Source |
| --- | --- | --- |
| Mediating indicators |  |  |
| Social assets |  |  |
| Self-efficacy | Score with range 0-10 equal to the sum of agree responses to the following ten agree/disagree items:   1. I can always manage to solve difficult problems if I try hard enough. 2. If someone is against me, I can still find ways to get what I want. 3. It is easy for me to focus on my aims and accomplish my goals. 4. I am confident that I could handle unexpected events very well. 5. Because of the help I can get, I know how to manage unexpected situations. 6. I can solve most problems if I make the necessary effort. 7. I can remain calm when facing difficulties because I can rely on my own abilities. 8. When I face a problem, I can usually find more than one solution. 9. If I am in trouble, I can usually think of a solution. 10. I can usually handle any situation that comes my way.   Baseline alpha = 0.8163 | Generalized Self-Efficacy Scale (Schwarzer & Jerusalem 1995) |
| Had a safe space in community to meet with friends | Binary variable equal to 1 if agreed with the following statement:  There is a place in the community, other than your house, your friend’s house or school, where you feel safe to meet your girl friends. |  |
| Positive gender attitudes | Score with range 0-7 equal to the sum of positive gender attitudes responses to the following seven agree/disagree items:   1. Males are better at managing money than females. (1 point if disagree) 2. Fathers in the family should decide on how family money is spent. (1 point if disagree) 3. It is as important for girls to complete secondary school as it is for boys. (1 point if agree) 4. When a family cannot afford to send all children to school, it is better to send boys than girls. (1 point if disagree) 5. When a husband and wife disagree about the number of children to have, the husband's opinion matters more. (1 point if disagree) 6. A girl should get married when she finds an appropriate partner, even if she is still in school. (1 point if disagree) 7. Girls are as intelligent as boys. (1 point if agree)   Baseline alpha = 0.4725 |  |
| Non-acceptability of IPV | Binary variable equal to 1 if responded that a husband is not justified in hitting or beating his wife in each of the following five situations:   1. If she goes out without telling him 2. If she neglects the children 3. If she argues with him 4. If she refuses to have sex with him 5. If she burns the food | Demographic and Health Survey |
| Economic assets |  |  |
| Financial literacy | Score with range 0-9 equal to the sum of correct responses to the following nine items:  "Each week, Anna Phiri sits down and plans what she will earn and spend in the next week. She writes down all the places where she will get money and all the things she will spend it on. Then she is able to see if she has enough money for all of what she wants to buy."   1. What would you call that kind of plan?   "Chilombo is 17 and has a sister, Pamela, who is married and lives in another town. Pamela just had a baby boy, and Chilombo is eager to visit her sister. Chilombo will need to save money for transport and a small hat for the baby. But she can’t take money from her savings because she is saving that money to start her own business. Hopefully, her neighbor will employ her to work extra days in her lodge so she can get the money she needs for her trip."   1. What is one of Chilombo's short term financial goals? 2. What is Chilombo’s long term financial goal? 3. What is one formal way of saving your money? 4. What is one informal way of saving your money? 5. Grace would like to buy a new notebook for the next school term which starts in eight weeks. If the notebook costs KR 10 and she can save KR 1 each week, will she reach her goal? 6. In the situation I described in the previous question, if Grace figured out how much she needed to save each week, and for how many weeks she needed to save in order to reach her goal, what would that be called? 7. If Grace discovered that she couldn’t reach her goal with that plan, what changes could she make so she would still reach her goal? 8. Do you agree or disagree with the following statement: “Only people with a lot of money can save”?   Baseline alpha: 0.6236 |  |
| Saved money in the past year | Binary variable equal to 1 if responded yes to the following question:  Have you saved, or put money aside to use at a later time, in the past year? |  |
| Health assets |  |  |
| Fertile period and contraceptive methods knowledge | Score with range 0-11 equal to the sum of correct responses to two questions about the fertile period and spontaneous mention of up to nine family planning methods listed below:   1. From one menstrual period to the next, are there certain days when a woman is more likely to become pregnant if she has sexual relations? 2. Is this time just before her period begins, during her period, right after her period has ended, or two weeks after her period? 3. Spontaneous mention of method: Pill 4. Spontaneous mention of method: IUD 5. Spontaneous mention of method: Injectables 6. Spontaneous mention of method: Implants 7. Spontaneous mention of method: Male condom 8. Spontaneous mention of method: Female condom 9. Spontaneous mention of method: Rhythm method 10. Spontaneous mention of method: Withdrawal 11. Spontaneous mention of method: Emergency contraception   Baseline alpha: 0.7718 | Demographic and Health Survey |
| HIV knowledge | Score with range 0-11 equal to the sum of responses indicating awareness and knowledge of HIV/AIDS from the following eleven items:   1. Have you ever heard of an illness called AIDS? 2. Can people reduce their chances of getting the AIDS virus by having just one uninfected sex partner who has no other sex partners? 3. Can people get the AIDS virus from mosquito bites? 4. Can people reduce their chance of getting the AIDS virus by using a condom every time they have sex? 5. Can people get the AIDS virus by sharing food with a person who has AIDS? 6. Can people reduce their chance of getting the AIDS virus by not having sexual intercourse at all? 7. Can people get the AIDS virus because of witchcraft or other supernatural means? 8. Is it possible for a healthy-looking person to have the AIDS virus? 9. Can the virus that causes AIDS be transmitted from a mother to her baby during pregnancy? 10. Can the virus that causes AIDS be transmitted from a mother to her baby during delivery? 11. Can the virus that causes AIDS be transmitted from a mother to her baby by breastfeeding?   Baseline alpha: 0.8969 | Demographic and Health Survey |
| Health assets |  |  |
| Used condom at first sex | Binary variable equal to 1 if responded yes to the following question:  The first time you had sexual intercourse, was a condom used? |  |
| Agreed to having had transactional sex | Binary variable equal to 1 if agreed with any of the following three statements:   1. You have had sex with a person in exchange for protection or a place to stay. 2. You have had sex with a person in exchange for him paying rent for you. 3. You have had sex because you thought you would get money. |  |
| Longer-term outcomes |  |  |
| Education |  |  |
| Completed grade 7 | Binary variable equal to 1 if had completed grade 7 (the last grade of primary school) determined from the following questions:  What is the highest level of school you attended: primary, secondary, or higher (college/university)?  What is the highest grade you completed at primary school? |  |
| Completed grade 9 | Binary variable equal to 1 if had completed grade 9 (junior secondary school) determined from the following questions:  What is the highest level of school you attended: primary, secondary, or higher (college/university)?  What is the highest grade you completed at secondary school? |  |
| Fertility (among girls 15 and older) |  |  |
| Ever married | Binary variable equal to 1 if had ever been married or lived with someone as if married determined from the following question:  Have you ever been married or lived with someone as if married? |  |
| Ever had sex | Binary variable equal to 1 if had ever had sex determined from the following questions:  Have you ever had sexual intercourse with a boyfriend?  Have you ever had sexual intercourse with a "sugar daddy"?  Have you ever had sexual intercourse with a casual acquaintance?  Have you ever had sexual intercourse with a relative?  Have you ever had sexual intercourse with a teacher?  How old were you when you had sexual intercourse for the very first time?  You indicated that you do not know how old you were when you had sexual intercourse for the first time. By sexual intercouse, we mean when a man inserts his penis into a woman's vagina. Just to be sure, have you ever had sexual intercourse? |  |
| Ever pregnant | Binary variable equal to 1 if had ever been pregnant determined from the following questions:  Have you ever given birth?  Have you ever given birth to a boy or girl who was born alive but later died?  Are you pregnant now?  Have you ever had a pregnancy that miscarried, was aborted, or ended in a stillbirth?  Sometimes a girl becomes pregnant when she does not want to be. Have you ever been pregnant when you did not want to be? |  |
| Ever given birth | Binary variable equal to 1 if had ever given birth determined from the following questions:  Have you ever given birth?  Have you ever given birth to a boy or girl who was born alive but later died? |  |
