# Supplementary material for: The impact of the Adolescent Girls Empowerment Program (AGEP) on short and long term social, economic, education and fertility outcomes: a cluster randomized controlled trial in Zambia
Source: BMC Public Health. 2020 Mar 17;20:349. doi: 10.1186/s12889-020-08468-0 (PMC7079524; doi:10.1186/s12889-020-08468-0)
Supplement: Supplementary file 2 — Additional file 2. Results from a probit regression for attrition. [file 12889_2020_8468_MOESM2_ESM.docx]

**Appendix 2:** Results from a probit regression for attrition

|  | Coef |  | 95% CI | |
| --- | --- | --- | --- | --- |
| Study arms |  |  |  |  |
| Arm 1 | 0.056 |  | -0.050 | 0.162 |
| Arm 2 | -0.014 |  | -0.117 | 0.090 |
| Arm 3 | 0.024 |  | -0.084 | 0.133 |
| Control arm (ref) |  |  |  |  |
| Age | 0.030 | * | 0.004 | 0.055 |
| Had attended current school year | -0.129 | * | -0.240 | -0.018 |
| Highest grade completed | -0.041 | ** | -0.068 | -0.013 |
| Literate | -0.002 |  | -0.098 | 0.095 |
| Mother's living and co-residence status |  |  |  |  |
| Co-resident with girl (ref) |  |  |  |  |
| Alive but not co-resident with girl | 0.358 | *** | 0.263 | 0.454 |
| Not alive | 0.047 |  | -0.077 | 0.170 |
| Father's living and co-residence status |  |  |  |  |
| Co-resident with girl (ref) |  |  |  |  |
| Alive but not co-resident with girl | 0.051 |  | -0.052 | 0.154 |
| Not alive | 0.003 |  | -0.107 | 0.112 |
| Mother completed primary school | -0.032 |  | -0.111 | 0.046 |
| Father completed primary school | -0.039 |  | -0.130 | 0.051 |
| Household wealth quintiles |  |  |  |  |
| 1 Poorest | 0.049 |  | -0.075 | 0.173 |
| 2 | 0.129 | † | -0.002 | 0.259 |
| 3 | 0.100 |  | -0.030 | 0.230 |
| 4 | 0.008 |  | -0.115 | 0.131 |
| 5 Wealthiest (ref) |  |  |  |  |
| Vulnerability quintiles |  |  |  |  |
| 1 Lowest vulnerability (ref) |  |  |  |  |
| 2 | -0.009 |  | -0.128 | 0.110 |
| 3 | -0.017 |  | -0.133 | 0.100 |
| 4 | 0.131 | † | -0.001 | 0.263 |
| 5 Highest vulnerability | 0.107 |  | -0.038 | 0.251 |
| Number of girls | 4661 |  |  |  |
| Wald chi2 | 204.21 | *** |  |  |
| All covariates measured at baseline. Model also included dummies for study sites. | | |  |  |
| Robust standard errors adjusted for clusters at the CSA level. | |  |  |  |
| *** p<0.001, ** p<0.01, * p<0.05, † p<0.1 |  |  |  |  |
