# Supplementary material for: The impact of the Adolescent Girls Empowerment Program (AGEP) on short and long term social, economic, education and fertility outcomes: a cluster randomized controlled trial in Zambia
Source: BMC Public Health. 2020 Mar 17;20:349. doi: 10.1186/s12889-020-08468-0 (PMC7079524; doi:10.1186/s12889-020-08468-0)
Supplement: Supplementary file 3 — Additional file 3. Estimated difference-in-differences (DID) for intent-to-treat (ITT) by intervention arm, results from linear regressions with girl-level fixed effects. [file 12889_2020_8468_MOESM3_ESM.docx]

**Appendix 3:** Estimated difference-in-differences (DID) for intent-to-treat (ITT) by intervention arm, results from linear regressions with girl-level fixed effects

|  | Arm 1 | | | | Arm 2 | | | | Arm 3 | | | |
| --- | --- | --- | --- | --- | --- | --- | --- | --- | --- | --- | --- | --- |
|  | DID coef |  | 95% CI | | DID coef |  | 95% CI | | DID coef |  | 95% CI | |
| Social assets |  |  |  |  |  |  |  |  |  |  |  |  |
| Self-efficacy score [0-10] |  |  |  |  |  |  |  |  |  |  |  |  |
| Round 3 | -0.002 |  | -0.339 | 0.335 | 0.159 |  | -0.174 | 0.491 | 0.144 |  | -0.182 | 0.470 |
| Round 5 | 0.203 |  | -0.134 | 0.540 | 0.280 |  | -0.075 | 0.635 | 0.447 | * | 0.099 | 0.796 |
| Had a safe space in community to meet with friends |  |  |  |  |  |  |  |  |  |  |  |  |
| Round 3 | 0.094 | * | 0.008 | 0.180 | 0.084 | * | 0.010 | 0.158 | 0.067 | † | -0.006 | 0.140 |
| Round 5 | 0.051 |  | -0.023 | 0.126 | 0.001 |  | -0.071 | 0.073 | -0.017 |  | -0.092 | 0.057 |
| Positive gender attitudes score [0-7] |  |  |  |  |  |  |  |  |  |  |  |  |
| Round 3 | -0.067 |  | -0.298 | 0.165 | 0.007 |  | -0.233 | 0.247 | 0.029 |  | -0.196 | 0.255 |
| Round 5 | -0.044 |  | -0.305 | 0.217 | 0.093 |  | -0.190 | 0.376 | -0.043 |  | -0.314 | 0.227 |
| Non-acceptability of IPV |  |  |  |  |  |  |  |  |  |  |  |  |
| Round 3 | -0.062 | † | -0.134 | 0.010 | 0.000 |  | -0.076 | 0.077 | 0.042 |  | -0.035 | 0.118 |
| Round 5 | -0.028 |  | -0.107 | 0.052 | 0.032 |  | -0.046 | 0.110 | 0.017 |  | -0.061 | 0.094 |
| Economic assets |  |  |  |  |  |  |  |  |  |  |  |  |
| Financial literacy score [0-9] |  |  |  |  |  |  |  |  |  |  |  |  |
| Round 3 | 0.195 |  | -0.085 | 0.474 | 0.238 |  | -0.075 | 0.551 | 0.297 | * | 0.045 | 0.549 |
| Round 5 | 0.216 |  | -0.104 | 0.536 | 0.150 |  | -0.179 | 0.480 | 0.263 | † | -0.039 | 0.565 |
| Saved money in the past year |  |  |  |  |  |  |  |  |  |  |  |  |
| Round 3 | 0.030 |  | -0.031 | 0.092 | 0.074 | * | 0.011 | 0.136 | 0.096 | ** | 0.034 | 0.159 |
| Round 5 | 0.042 |  | -0.020 | 0.103 | 0.071 | * | 0.008 | 0.135 | 0.086 | ** | 0.026 | 0.147 |
| Health assets |  |  |  |  |  |  |  |  |  |  |  |  |
| Fertile period and contraceptive methods knowledge score [0-11] | |  |  |  |  |  |  |  |  |  |  |  |
| Round 3 | 0.326 | * | 0.070 | 0.582 | 0.338 | ** | 0.116 | 0.559 | 0.202 | † | -0.035 | 0.439 |
| Round 5 | 0.281 | * | 0.002 | 0.559 | 0.206 |  | -0.057 | 0.469 | 0.320 | * | 0.056 | 0.585 |
| HIV knowledge score [0-11] |  |  |  |  |  |  |  |  |  |  |  |  |
| Round 3 | 0.165 |  | -0.292 | 0.621 | 0.134 |  | -0.281 | 0.549 | 0.021 |  | -0.439 | 0.482 |
| Round 5 | 0.185 |  | -0.260 | 0.630 | 0.100 |  | -0.326 | 0.525 | 0.037 |  | -0.441 | 0.515 |
| Sexual behavior among girls ages 15 and older who had ever had sex | | |  |  |  |  |  |  |  |  |  |  |
| Used condom at first sex^a^ |  |  |  |  |  |  |  |  |  |  |  |  |
| Round 3 | 0.055 |  | -0.053 | 0.163 | 0.019 |  | -0.087 | 0.125 | 0.068 |  | -0.044 | 0.179 |
| Round 5 | 0.014 |  | -0.069 | 0.096 | -0.013 |  | -0.089 | 0.064 | 0.028 |  | -0.048 | 0.104 |
| Agreed to having had transactional sex |  |  |  |  |  |  |  |  |  |  |  |  |
| Round 3 | -0.126 | † | -0.272 | 0.019 | -0.128 | † | -0.264 | 0.007 | -0.104 |  | -0.246 | 0.038 |
| Round 5 | -0.122 | † | -0.267 | 0.022 | -0.137 | * | -0.263 | -0.012 | -0.095 | † | -0.205 | 0.016 |
| Education outcomes |  |  |  |  |  |  |  |  |  |  |  |  |
| Completed grade 7 |  |  |  |  |  |  |  |  |  |  |  |  |
| Round 3 | -0.015 |  | -0.059 | 0.029 | 0.016 |  | -0.033 | 0.064 | -0.014 |  | -0.059 | 0.032 |
| Round 5 | 0.020 |  | -0.034 | 0.074 | 0.019 |  | -0.035 | 0.074 | 0.013 |  | -0.039 | 0.064 |
| Completed grade 9 |  |  |  |  |  |  |  |  |  |  |  |  |
| Round 3 | -0.007 |  | -0.051 | 0.036 | 0.012 |  | -0.032 | 0.055 | -0.010 |  | -0.051 | 0.032 |
| Round 5 | -0.017 |  | -0.072 | 0.038 | -0.011 |  | -0.069 | 0.047 | -0.016 |  | -0.069 | 0.037 |
| Fertility outcomes among girls ages 15 and older |  |  |  |  |  |  |  |  |  |  |  |  |
| Ever married |  |  |  |  |  |  |  |  |  |  |  |  |
| Round 3 | 0.026 |  | -0.040 | 0.092 | 0.026 |  | -0.042 | 0.094 | -0.019 |  | -0.087 | 0.048 |
| Round 5 | 0.017 |  | -0.068 | 0.102 | 0.034 |  | -0.058 | 0.125 | 0.019 |  | -0.078 | 0.116 |
| Ever had sex |  |  |  |  |  |  |  |  |  |  |  |  |
| Round 3 | 0.054 | † | -0.008 | 0.115 | 0.052 |  | -0.017 | 0.121 | 0.025 |  | -0.046 | 0.096 |
| Round 5 | 0.081 | * | 0.007 | 0.155 | 0.083 | * | 0.012 | 0.153 | 0.043 |  | -0.029 | 0.114 |
| Ever pregnant |  |  |  |  |  |  |  |  |  |  |  |  |
| Round 3 | -0.014 |  | -0.095 | 0.068 | -0.004 |  | -0.080 | 0.072 | -0.021 |  | -0.094 | 0.053 |
| Round 5 | 0.020 |  | -0.064 | 0.104 | 0.038 |  | -0.040 | 0.117 | 0.041 |  | -0.037 | 0.120 |
| Ever given birth |  |  |  |  |  |  |  |  |  |  |  |  |
| Round 3 | 0.003 |  | -0.073 | 0.079 | 0.005 |  | -0.073 | 0.083 | 0.010 |  | -0.064 | 0.084 |
| Round 5 | 0.023 |  | -0.056 | 0.102 | 0.035 |  | -0.048 | 0.117 | 0.026 |  | -0.052 | 0.103 |
| All models adjust for age. Robust standard errors adjusted for clusters at the CSA level. | |  |  |  |  |  |  |  |  |  |  |  |
| *** p<0.001, ** p<0.01, * p<0.05, † p<0.1 |  |  |  |  |  |  |  |  |  |  |  |  |
| ^a^ Estimated as simple differences at each round between intervention and control arms excluding girls who had ever had sex at baseline and adjusting for age and study site. | | | | | | | | | | | |  |
