# Supplementary material for: The impact of the Adolescent Girls Empowerment Program (AGEP) on short and long term social, economic, education and fertility outcomes: a cluster randomized controlled trial in Zambia
Source: BMC Public Health. 2020 Mar 17;20:349. doi: 10.1186/s12889-020-08468-0 (PMC7079524; doi:10.1186/s12889-020-08468-0)
Supplement: Supplementary file 4 — Additional file 4. Estimated difference-in-differences (DID) for treatment-on-the-treated (TOT) by intervention arms, results from two-stage least squares IV regressions with girl-level fixed-effects. [file 12889_2020_8468_MOESM4_ESM.docx]

**Appendix 4:** Estimated difference-in-differences (DID) for treatment-on-the-treated (TOT) by intervention arms, results from two-stage least squares IV regressions with girl-level fixed-effects

|  | Arm 1 | | | | Arm 2 | | | | Arm 3 | | | |
| --- | --- | --- | --- | --- | --- | --- | --- | --- | --- | --- | --- | --- |
|  | DID coef |  | 95% CI | | DID coef |  | 95% CI | | DID coef |  | 95% CI | |
| Social assets |  |  |  |  |  |  |  |  |  |  |  |  |
| Self-efficacy score [0-10] |  |  |  |  |  |  |  |  |  |  |  |  |
| Round 3 | -0.005 |  | -0.973 | 0.963 | 0.433 |  | -0.482 | 1.348 | 0.439 |  | -0.543 | 1.420 |
| Round 5 | 0.585 |  | -0.376 | 1.547 | 0.765 |  | -0.226 | 1.756 | 1.357 | * | 0.324 | 2.391 |
| Had a safe space in community to meet with friends |  |  |  |  |  |  |  |  |  |  |  |  |
| Round 3 | 0.271 | * | 0.015 | 0.528 | 0.231 | * | 0.028 | 0.434 | 0.205 | † | -0.022 | 0.431 |
| Round 5 | 0.148 |  | -0.070 | 0.367 | 0.001 |  | -0.194 | 0.197 | -0.052 |  | -0.276 | 0.172 |
| Positive gender attitudes score [0-7] |  |  |  |  |  |  |  |  |  |  |  |  |
| Round 3 | -0.192 |  | -0.857 | 0.472 | 0.020 |  | -0.633 | 0.672 | 0.089 |  | -0.590 | 0.768 |
| Round 5 | -0.127 |  | -0.871 | 0.617 | 0.256 |  | -0.514 | 1.025 | -0.132 |  | -0.945 | 0.682 |
| Non-acceptability of IPV |  |  |  |  |  |  |  |  |  |  |  |  |
| Round 3 | -0.181 |  | -0.396 | 0.035 | 0.001 |  | -0.206 | 0.208 | 0.127 |  | -0.105 | 0.359 |
| Round 5 | -0.081 |  | -0.309 | 0.148 | 0.087 |  | -0.124 | 0.298 | 0.051 |  | -0.183 | 0.284 |
| Economic assets |  |  |  |  |  |  |  |  |  |  |  |  |
| Financial literacy score [0-9] |  |  |  |  |  |  |  |  |  |  |  |  |
| Round 3 | 0.563 |  | -0.234 | 1.361 | 0.655 |  | -0.207 | 1.517 | 0.899 | * | 0.131 | 1.667 |
| Round 5 | 0.622 |  | -0.284 | 1.529 | 0.410 |  | -0.480 | 1.300 | 0.795 | † | -0.116 | 1.706 |
| Saved money in the past year |  |  |  |  |  |  |  |  |  |  |  |  |
| Round 3 | 0.088 |  | -0.086 | 0.263 | 0.202 | * | 0.034 | 0.371 | 0.292 | ** | 0.098 | 0.485 |
| Round 5 | 0.120 |  | -0.052 | 0.293 | 0.195 | * | 0.020 | 0.371 | 0.262 | ** | 0.076 | 0.447 |
| Health assets |  |  |  |  |  |  |  |  |  |  |  |  |
| Fertile period and contraceptive methods knowledge score [0-11] | |  |  |  |  |  |  |  |  |  |  |  |
| Round 3 | 0.936 | * | 0.211 | 1.660 | 0.925 | ** | 0.292 | 1.557 | 0.618 | † | -0.102 | 1.337 |
| Round 5 | 0.802 | * | 0.035 | 1.569 | 0.559 |  | -0.169 | 1.287 | 0.974 | * | 0.158 | 1.790 |
| HIV knowledge score [0-11] |  |  |  |  |  |  |  |  |  |  |  |  |
| Round 3 | 0.476 |  | -0.827 | 1.779 | 0.367 |  | -0.761 | 1.495 | 0.065 |  | -1.320 | 1.451 |
| Round 5 | 0.535 |  | -0.722 | 1.791 | 0.272 |  | -0.880 | 1.423 | 0.111 |  | -1.320 | 1.543 |
| Sexual behavior among girls ages 15 and older who had ever had sex | |  |  |  |  |  |  |  |  |  |  |  |
| Used condom at first sex^a^ |  |  |  |  |  |  |  |  |  |  |  |  |
| Round 3 | 0.269 |  | -0.267 | 0.805 | 0.067 |  | -0.321 | 0.455 | 0.248 |  | -0.176 | 0.672 |
| Round 5 | 0.044 |  | -0.226 | 0.314 | -0.038 |  | -0.261 | 0.184 | 0.099 |  | -0.170 | 0.368 |
| Agreed to having had transactional sex |  |  |  |  |  |  |  |  |  |  |  |  |
| Round 3 | -0.533 | † | -1.152 | 0.087 | -0.581 | † | -1.213 | 0.051 | -0.463 |  | -1.108 | 0.181 |
| Round 5 | -0.514 |  | -1.156 | 0.129 | -0.618 | * | -1.209 | -0.027 | -0.420 |  | -0.931 | 0.091 |
| Education outcomes |  |  |  |  |  |  |  |  |  |  |  |  |
| Completed grade 7 |  |  |  |  |  |  |  |  |  |  |  |  |
| Round 3 | -0.043 |  | -0.170 | 0.083 | 0.043 |  | -0.090 | 0.176 | -0.041 |  | -0.178 | 0.095 |
| Round 5 | 0.057 |  | -0.097 | 0.212 | 0.052 |  | -0.096 | 0.201 | 0.038 |  | -0.115 | 0.192 |
| Completed grade 9 |  |  |  |  |  |  |  |  |  |  |  |  |
| Round 3 | -0.021 |  | -0.145 | 0.103 | 0.032 |  | -0.086 | 0.151 | -0.030 |  | -0.154 | 0.095 |
| Round 5 | -0.050 |  | -0.207 | 0.107 | -0.030 |  | -0.189 | 0.129 | -0.047 |  | -0.205 | 0.110 |
| Fertility outcomes among girls ages 15 and older |  |  |  |  |  |  |  |  |  |  |  |  |
| Ever married |  |  |  |  |  |  |  |  |  |  |  |  |
| Round 3 | 0.094 |  | -0.150 | 0.338 | 0.089 |  | -0.142 | 0.320 | -0.070 |  | -0.332 | 0.192 |
| Round 5 | 0.064 |  | -0.241 | 0.369 | 0.113 |  | -0.189 | 0.415 | 0.069 |  | -0.299 | 0.436 |
| Ever had sex |  |  |  |  |  |  |  |  |  |  |  |  |
| Round 3 | 0.201 | † | -0.020 | 0.422 | 0.177 |  | -0.059 | 0.414 | 0.099 |  | -0.174 | 0.372 |
| Round 5 | 0.293 | * | 0.032 | 0.555 | 0.274 | * | 0.031 | 0.518 | 0.163 |  | -0.115 | 0.440 |
| Ever pregnant |  |  |  |  |  |  |  |  |  |  |  |  |
| Round 3 | -0.048 |  | -0.345 | 0.249 | -0.010 |  | -0.262 | 0.241 | -0.072 |  | -0.353 | 0.208 |
| Round 5 | 0.068 |  | -0.232 | 0.368 | 0.125 |  | -0.134 | 0.384 | 0.152 |  | -0.148 | 0.452 |
| Ever given birth |  |  |  |  |  |  |  |  |  |  |  |  |
| Round 3 | 0.012 |  | -0.263 | 0.287 | 0.020 |  | -0.241 | 0.281 | 0.042 |  | -0.240 | 0.325 |
| Round 5 | 0.083 |  | -0.202 | 0.367 | 0.114 |  | -0.159 | 0.387 | 0.097 |  | -0.200 | 0.393 |
| All models adjust for age. Robust standard errors adjusted for clusters at the CSA level. |  |  |  |  |  |  |  |  |  |  |  |  |
| *** p<0.001, ** p<0.01, * p<0.05, † p<0.1 |  |  |  |  |  |  |  |  |  |  |  |  |
| ^a^ Estimated as simple differences at each round between intervention and control arms excluding girls who had ever had sex at baseline and adjusting for age and study site. | | | | | | | | | |  |  |  |
